# Analysis of Criteria for MRI Diagnosis of TMJ Disc Displacement and Arthralgia

**DOI:** 10.1155/2012/283163

**Published:** 2012-12-09

**Authors:** Jeffry R. Shaefer, Cara Joy Riley, Paul Caruso, David Keith

**Affiliations:** ^1^Department of Oral and Maxillofacial Surgery, Harvard School of Dental Medicine, 4 Monument Circle, Hingham, MA 02043, USA; ^2^Children's Hospital Colorado, 13123 E 16th Avenue B090, Aurora, CO 80045, USA; ^3^Department of Radiology, Massachusetts General Hospital, Massachusetts Eye and Ear Infirmary, 243 Charles Street, Boston, MA 02114, USA; ^4^Department of Oral and Maxillofacial Surgery, Massachusetts General Hospital, Warren Building Suite 1201, 55 Fruit Street, Boston, MA 02114, USA

## Abstract

*Aims*. To improve diagnostic criteria for TMJ disc displacement (DD). *Methods*. The standard protocol for MRI diagnosis of DD, using a 12 o'clock reference position, was compared to an alternative protocol. The alternative protocol involves the functional relationship between the condyle and articular eminence, using a line perpendicular to the posterior slope of the eminence as a reference for disc position. The disc location was examined using both protocols, and disc diagnoses were compared in their relationship with joint pain. Statistical analyses included *P* value, sensitivity, specificity, odds ratio, and kappa statistic. *Results*. 58 MRIs were interpreted. 36 subjects reported arthralgia; 22 did not. Both protocols demonstrated significance (standard *P* = 0.004, alternative *P* < 0.001) for the ability to predict arthralgia. The odds of arthralgia increased in DD patients diagnosed by standard methods 9.71 times and in DD diagnosed by alternative means 37.15 times. The diagnostic sensitivity decreased 30% using the alternative versus the standard protocol (0.6389 versus 0.9444), while specificity increased 60% (0.9545 versus 0.3636). *Conclusions*. A stronger relationship occurs between DD and arthralgia when using a function-based protocol. The alternative protocol correctly identifies subjects without arthralgia, who by standard methods would be diagnosed with DD, as having nondisplaced discs, providing a more clinically relevant assessment of TMJ disc displacement.

## 1. Introduction

### 1.1. Background and Significance

Temporomandibular joint pain is considered to develop as the result of inflammatory and/or mechanical mechanisms [1]. Gross morphological changes such as deviation in form, disc displacement, adhesions, and osteoarthritic processes can occur with or without the subject's perceiving pain or dysfunction [2]. When do such findings relate to pain and dysfunction? In Westesson's summary regarding the imaging diagnosis of TMJ arthralgia he states, “inflammatory changes correlate strongly with the patient's pain symptoms [and] we are getting closer to imaging the changes that are truly relevant to [these] symptoms” [3]. In his study, he focused on the most symptomatic TMJ in each subject, but found a high number of abnormalities consistent with disc displacement and inflammation in the contralateral joint, as well. These asymptomatic TMJ imaging abnormalities are reminiscent of asymptomatic meniscal and lumbar disc abnormalities often seen in knee and back patients, respectively. This similar imaging conundrum supports the concept that preexisting TMJ disc degeneration can predispose a patient to traumatic or spontaneous symptomatic disease. Researchers at the National Institute of Health lamented such vagaries of TMD, stating “jaw joint pain is a billion-dollar problem in the United States and medical science is still uncertain how to fix it” [4].

At what point do alterations in the TMJ, such as disc displacement, progress from adaptive changes to dysfunctional and/or pathological changes? An accepted sign of TMJ dysfunction (pain and limited range-of-motion (ROM)) is the presence of inflammation. Effusions found on TMJ MRIs are commonly attributed to inflammation and can often be considered an indication for TMJ surgery. Shaefer found, however, in his cohort of DD with reduction subjects that the presence of MRI effusions was not associated with arthralgia [5]. In Manfredini's study TMJ effusions on MRI and arthralgia were associated in subjects who had DD without reduction but not DD with reduction [6]. A number of authors have alluded that TMJ dysfunction (pain and limited range-of-motion (ROM)) is not related to disc displacement [1, 7–12]. Does a functionally based protocol for determining disc position in the sagittal plane demonstrate a stronger relationship between disc displacement with reduction, arthralgia, and effusions?

MRI is considered the most effective imaging tool (gold standard) for evaluation of the TMJ soft tissues, the disc-condyle relationship, and for determination of disc displacement. The standard protocol for MRI diagnosis of anterior disc displacement uses the most superior surface (12 o'clock position) of the condyle as a reference point for the posterior band of the disk. A posterior band of the disk located anterior to the 12 o'clock position correlates to anterior disk displacement [13]. A disk or posterior band of a disc posterior to this reference point places the disk posterior to the functional area of the TMJ. The anterior-superior part of the condyle and the posterior slope of the articular eminence are acknowledged as the functional areas on the articular surfaces of the TMJ [14]. Rammelsberg described an alternative technique for determining TMJ disc displacement that uses this functional anterior-superior part of the condyle as the reference position for normal disc position [15]. In his study, Rammelsberg used the standard protocol to analyze sagittal MRI slices for variability of disc position in the TMJ in the coronal plane medially laterally. He discovered that subjects with symptomatic disc displacement (e.g., clicking and/or restricted range of motion) averaged 77 degrees of disk displacement. Subjects with asymptomatic joints also had disk displacement, but to a lesser degree, averaging 15–30 degrees of displacement, but this milder displacement was located in the lateral portion of the joint. Such mild anterior displacement was concluded to be a variant of normal, a concept supported by other authors [5, 9, 16]. He concluded that a more profound anterior disk displacement is required for TMJ dysfunction and that this “advanced” disk placement occurs more in the lateral portion of the joint due to the oblique functional alignment of the long axis of the condyle; that is, the lateral pole of the condyle is positioned anterior to the medial pole of the condyle head. Such correlations suggest the need for an improved technique by which to determine TMJ disc displacement.

A functionally based alternative protocol for the MRI diagnosis of TMJ anterior disc displacement (ADD) is the basis of this study. The new reference point represented by this alternative protocol is based on a more functional orientation, so that the ADD diagnosis requires more complete displacement. We propose that determining disc displacement by using functional criteria will clarify the diagnosis of disk displacement, better delineate the association between TMJ disk displacement and arthralgia, and enhance our understanding of disc displacement in the pathophysiology of TMJ dysfunction.

## 2. Materials and Methods

The research protocol was approved by both the Harvard Medical School and Massachusetts General Hospital (MGH) Institutional Review Boards.

### 2.1. Study Design

We investigated the relationship of TMJ arthralgia and internal derangement in subjects with anterior disc displacement with reduction, using a new (alternative) and standard protocol for determining disc displacement in the TMJ. Using bilateral MRIs, a determination of disc position was made, once according to the standard protocol and once according to the alternative protocol, for each MRI analyzed. In the standard protocol the criteria for the determination of anterior disc displacement in the TMJ are a posterior band of the disc that lies anterior to the 12 o'clock position on the mandibular condyle. The alternative protocol uses the point on the superior surface of the condyle that intersects with a plane perpendicular to the posterior slope of the articular eminence of the temporal bone (the TMJ fossa) as a reference point for the posterior band of the disc. The tangent for the slope of the eminence is determined by a line drawn between the 12 o'clock position of the glenoid fossa and the most inferior posterior point of the base of the articular eminence. The perpendicular line to this tangent is taken from the midpoint of the distance between the 12 o'clock position of the glenoid fossa and the most inferior posterior point of the base of the articular eminence. A posterior band anterior to this point is considered indicative of an anteriorly displaced disc (see Figure 2).

### 2.2. Methods

There were two main investigators in the study, the research mentor and the student researcher, who interpreted the MRI images together. Before the study began, the student underwent a reliability study to be trained in reading MRIs with two board certified members of the Department of Oral and Maxillofacial Surgery, Massachusetts General Hospital in order to standardize the interpretation of disc position on MRIs. During the interpretation of the MRIs, the examiner was blinded to the subjects' treatment records and history of TMJ arthralgia.

### 2.3. Study Sample

We studied the MRIs of 58 TMJs from the data pool at the University of Minnesota TIRR (TMJ Implant Registry and Repository) and the Orofacial Pain Clinic at Massachusetts General Hospital. The subjects met the criteria of anterior disc displacement with reduction, either by prior diagnosis from a radiologist or oral surgeon or as defined by the Research Diagnostic Criteria for TMD (RDC/TMD), an internationally recognized diagnostic system that has been shown to be reliable [17] and recently validated [18]. The presence of TMJ arthralgia was established via the treatment records of study participants. A subject was classified as arthralgia positive if he or she self-reported TMJ pain of at least three-month duration, with pain present on palpation and movement of the joint.

#### 2.3.1. All Subjects: Inclusion Criteria

1. Research Diagnostic Criteria for TMD, diagnosis of disc displacement with reduction.
  - Passive opening greater than or equal to 40 mm.
  - Has a clicking noise in the TMJ with jaw movement that meets one of the following criteria.
    - Reciprocal clicking that is reproducible in two of three consecutive trials. The click on both vertical opening and closing occurs at a point at least 5 mm greater interincisal distance on opening than on closing and is eliminated on protrusive opening.
    - Click on both vertical opening and closing and during lateral or protrusive excursions. These clicks are reproducible on two of three consecutive trials.
2. And/or
3. diagnosis by a radiologist and/or oral and maxillofacial surgeon of anterior disc displacement with reduction.

#### 2.3.2. TMJ Arthralgia: Inclusion Criteria

1. Self-report of jaw pain of at least three-month duration.
2. Pain in one or both joint sites during palpation of the TMJ.
3. One or more reports of pain in the region of the joint, pain in the joint during maximum unassisted opening, pain in the joint during assisted opening, and/or pain in the joint during lateral excursion.

#### 2.3.3. Adequate MRI Imaging Data

This study involved the MRI images of 58 TMJs and the corresponding treatment records for the subjects. The images obtained included the sagittal open and closed jaw positions. The MRI of interest for diagnosing anterior disc displacement was the central cut of the TMJ in the sagittal plane of the closed jaw position. The articular disc was directly identified as a biconcave area of hypointensity above the condyle, and its position was categorized as normal or anteriorly displaced.

### 2.4. Study Variables

#### 2.4.1. Independent

**(1) Diagnostic Protocols**

MRI diagnosis of anterior disc displacement with reduction was made using a new (alternative) protocol and using the standard protocol. The alternative protocol diagnosis was performed by drawing three lines: (a) the tangent to the slope of the articular eminence, (b) the perpendicular to that line, and (c) the tangent to the posterior band of the articular disc. The angle between (b) and (c) was then measured. An angle greater than zero degrees was recorded as anterior disc displacement. The standard protocol diagnosis was performed by drawing two lines: (a) the 12 o'clock line through the condyle and (b) the tangent to the posterior band of the articular disc. The angle between these two lines was measured. An angle greater than zero degrees was recorded as anterior disc displacement.

#### 2.4.2. Dependent

**(1) Diagnosis**

The presence or absence of anterior disc displacement.

**(2) Arthralgia**

Subjects who carry a prior diagnosis (outside this study) of anterior disc displacement with reduction and report arthralgia and those who carry a prior diagnosis of anterior disc displacement with reduction and have no report of arthralgia.

### 2.5. Data Analyses

Statistical analyses were completed using STATA software and Microsoft Excel 2007 for Windows XP. The student researcher performed the analysis, and Dr. Rachel Badovinac, DMD ScD (Harvard School of Dental Medicine), reviewed the calculations. The following two-by-two tables were created.

- Exposure: standard protocol ADD (presence or absence), outcome: arthralgia (presence or absence).
- Exposure: alternative protocol ADD (presence or absence), outcome: arthralgia (presence or absence).
- Exposure: ADD (standard or alternative protocol), outcome: arthralgia (presence or absence).
- Exposure: alternative protocol ADD (presence or absence), outcome: standard protocol ADD (presence or absence).

The relationship of ADD to joint pain using both protocols for determining anterior disc displacement was determined using Tables 1(a), 1(b), 1(c), and 1(d).

These tables were used to compare diagnoses from the standard and alternative protocols and the relationship of these diagnoses to the presence of arthralgia. More specifically, calculations of sensitivity, specificity, odds ratio (OR), and *P* value (significance at *P* < 0.05) were made. *P* values were determined by the Fisher's exact test due to small cell numbers in two of the cells of the 2 × 2 tables. Additionally, the kappa statistic was used to demonstrate the level of agreement between the two diagnostic protocols.

## 3. Results

The MRI images of 58 TMJs were interpreted using the central slice in the sagittal closed mouth position. The subjects were 8.67 : 1 female to male, with a mean age of 43 years. Thirty-six of the subjects reported arthralgia, while 22 did not. The determination of anterior disc displacement was recorded for each image, once using the standard protocol and once using the alternative protocol. The diagnosis as so determined was then related to the presence or absence of joint pain. The results are demonstrated in Tables 1(a)–1(d) (ADD: anterior disc displacement, STD: standard protocol, and ALT: alternative protocol).

The Fisher's exact test yielded a *P* value of 0.004 for the standard protocol and a *P* value of <0.001 for the alternative protocol. As such, both methods proved statistically significant for the ability of anterior disc displacement to identify joint pain.

Considering odds ratios (OR), the odds of arthralgia are increased 9.71 times in subjects diagnosed with ADD via the standard method versus those without the diagnosis via the standard method (OR = 9.71 [1.83, 51.60]). The equivalent calculation using the alternative protocol demonstrates 37.15 times increased odds of experiencing arthralgia in subjects with ADD (OR = 37.15 [4.47, 308.00]). The two confidence intervals do not contain the value 1.00 which agrees with the *P* values in terms of statistical significance.

The sensitivity of the diagnosis of ADD is increased for the standard (0.9444) versus the alternative (0.6389) protocol by approximately 30%. The strength of specificity is reversed, with an increase in specificity of approximately 60% for the alternative (0.9545) versus the standard (0.3636) methods of diagnosis. It is prudent to define the sensitivity and specificity further in this context, as the study does not contain the traditional “gold standard” outcome variable often associated with the terms. Rather, our study uses arthralgia as the outcome, and disc position as the exposure, and sensitivity and specificity are utilized simply to indicate the relationship between the two. As such, the sensitivity demonstrates the proportion of subjects who are diagnosed with ADD out of the total number with pain. Similarly, the specificity expresses the proportion of subjects who have normal disc position out of the total number without pain. Indeed, the direction of the relationship could be inverted, but we chose to focus on the ability of disc position to predict and identify arthralgia, not the reverse.

Comparing the two protocols side by side (Table 1(c)), the proportion of subjects experiencing arthralgia out of the total subjects with ADD is 1.35 higher for the alternative (96% or 23/24) versus the standard (71% or 34/48) protocol. Similarly, the proportion of those of not experiencing arthralgia is 7.00 times greater in those with ADD as diagnosed via standard (29% or 14/48) versus alternative (4% or 1/24) methods. This latter result is further substantiated by calculation of the type I error (false positive), at 64% (14/22) for the standard and 5% for the alternative protocols (1/22). However, of all subjects without the diagnosis of ADD, a higher proportion experienced arthralgia when diagnosed via alternative (38% or 13/34) as opposed to standard criteria (20% or 2/10). This result, in turn, is complemented by calculation of the type II error (false negative), at 36% (13/36) for the alternative and 6% (2/36) for the standard method.

### 3.1. Diagnostic Protocol Agreement

The concordance between the two diagnostic protocols was calculated using Table 1(d). The two protocols agreed on the diagnosis 59% percent ([24  +  10]/58) of the times. According to the standard protocol, anterior disc displacement was present in 83% (48/58) of TMJs, while the alternative protocol diagnosed the condition in 41% (24/58) of TMJs. The kappa statistic for agreement between the two protocols was 0.2564, where a value of 1.0000 represents perfect agreement. As such, the agreement between the standard and alternative protocols for anterior disc displacement can be categorized as mild to fair.

## 4. Discussion

**Figure 1:**
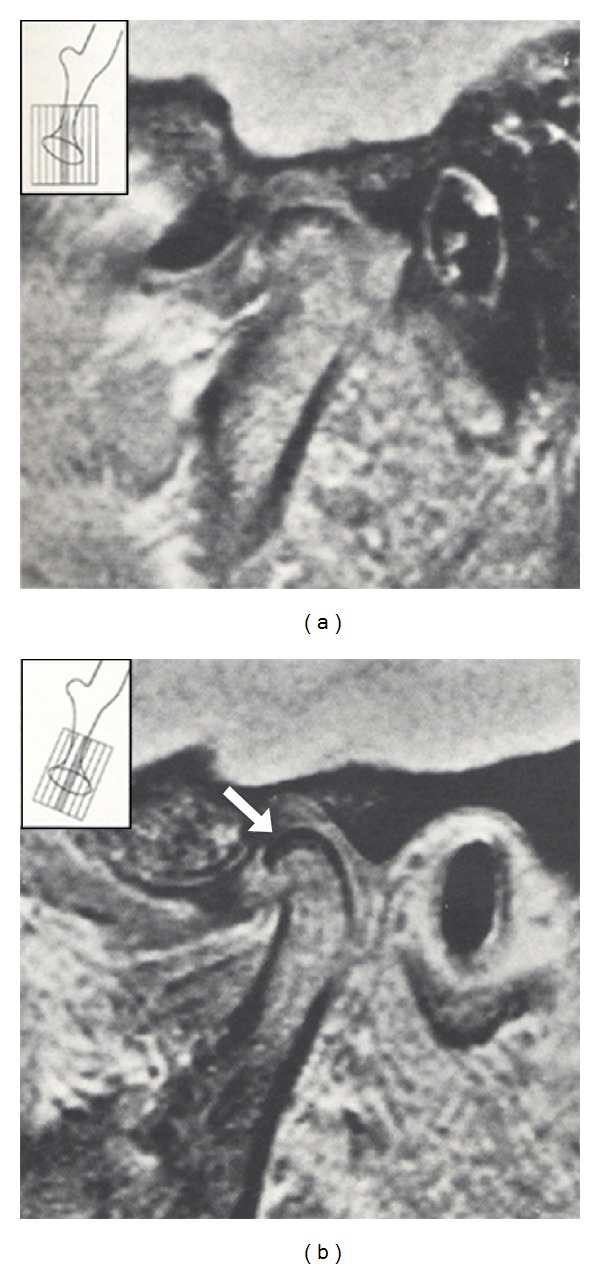
MRI images of the TMJ in the sagittal plane. MRI images in the central part of two joints, highlighting the sagittal versus the angulated planes. The image on the left shows the standard sagittal plane, and the posterior band of the disc is not visible. The image on the right shows the angulated plane of the same joint, and the posterior band is visible as indicated by the arrow. The insets in both images diagram the scanning planes [8].

**Figure 2:**
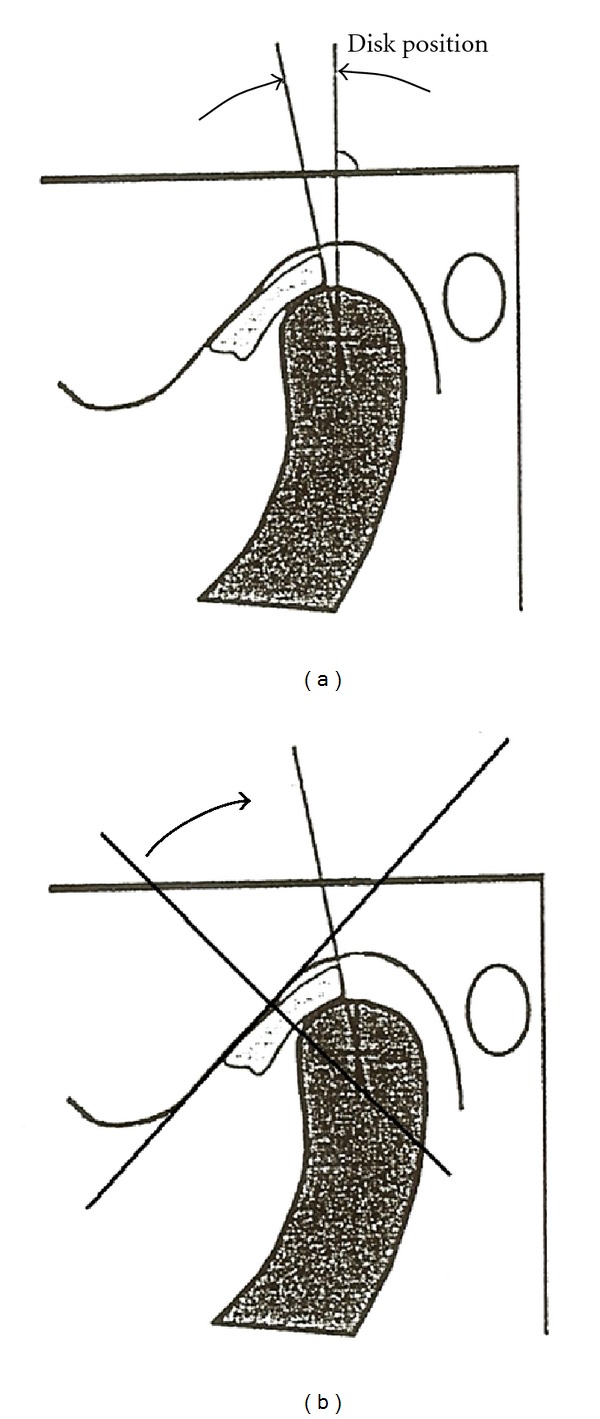
Protocols for diagnosis of anterior disc displacement. Angular measurement of disc position using the standard protocol (a) and the alternative protocol (b). In the standard protocol (on (a)), the posterior band of the disc is compared to the 12 o'clock position through the head of the condyle. In the alternative protocol (on (b)), the posterior band of the disc is compared to a line perpendicular to the tangent of the slope of the posterior eminence. The tangent used to represent the slope of the articular eminence was drawn superiorly between the 12 o'clock position at the most posterior part of the glenoid fossa of the temporal bone and anteriorly at the most inferior posterior point at the base of the articular eminence based on diagrams from [15].

Chin and Steenks each developed TMJ MRI protocols similar to the one presented in the current study to improve the identification of disc displacement and the correlation of TMJ imaging findings to clinical findings. Chen and Steenks [8, 19] proposed techniques to best orient direction of the imaging beam for a TMJ image. While Chen is concerned with coronal plane images to determine whether the disc is medially or laterally displaced, his concept is relevant for understanding anteriorly displaced discs in the sagittal plane. His study demonstrates that current imaging protocols for the TMJ can be improved, especially if these changes relate to the function of the TMJ. Chen recommends that rather than using a coronal slice parallel to a 90 degrees vertical to the condyle, coronal image slices parallel to a line perpendicular to the posterior slope of the articular eminence provide images more representative of medial or lateral disc displacement. The vertical taken at 90 degrees to the condyle shows the medial or lateral orientation of the posterior part of the disk compared to a line perpendicular to eminence which assesses orientation of the middle portion of the disk. Steenks, in keeping with this study, is concerned with the orientation of the sagittal image. Steenks showed that a scan angulated perpendicular to the long axis of the TMJ condylar head gives a better representation of the anterior-posterior position of the disc—particularly the posterior band—than an MRI scan taken from the standard sagittal view (refer to Figure 1). Chen and Steenks both apply functional parameters to enhance the technique for determining the degree of TMJ disc displacement.

An image corrected in the sagittal and coronal planes as suggested by Steenks and Chin would provide an image most accurately depicting the TMJ anatomy. In most parts of the world T joint MRIs are not taken in the true sagittal plane perpendicular to the long axis of the condylar head but taken at an estimated angle to be at right angles to the mandibular condyle. This would lead to a less accurate depiction of the TMJ anatomy on the image. That is true, but the image, when not corrected in a coronal plane to be perpendicular to the head of the condyle, will be parallel to the side of the face which does naturally correct the angle of the image to be somewhat perpendicular to the long axis of the condyle thereby improving the accuracy of the TMJ image.

A number of other studies probe the relationship between imaging evidence of disc displacement and clinical signs of joint dysfunction, such as arthralgia and clicking. Such studies reveal an agreement between the two of approximately 59%–90% [6, 20]. Our research confirms that there is agreement and seeks to enhance the agreement by changing the criteria for the imaging portion of the diagnosis. These criteria represent an improvement in the identification and classification of ADD patients.

According to the kappa statistic (0.2564), the standard and alternative protocols are discordant enough to consider the two separate and make it appropriate to compare the diagnoses imparted by each method. The results clearly indicate a relationship between TMJ pain and anterior disc displacement, diagnosed by either protocol (*P*-value of 0.004 for the standard and <0.001 for the alternative protocol). While both are statistically significant for identifying arthralgia in patients with ADD, the trend favors the alternative protocol. The OR demonstrates higher odds of arthralgia in ADD diagnosed by alternative criteria, although the wide range and overlapping of confidence intervals preclude a statement that one method is more significant than the other.

The specificity difference is where the distinction between the two protocols can be seen, with the alternative method demonstrating a 60% improvement over the standard in correctly identifying subjects without ADD as having an absence of joint pain (0.9545 versus 0.3636). In fact, the standard method of diagnosis classifies a higher proportion of subjects without arthralgia into the ADD category (64%) than the normal disc position category (36%). Accordingly, the standard criteria tend to “overdiagnose” ADD, naming subjects to the disc derangement group who lack signs of TMJ dysfunction. In terms of clinical relevance, the absence of false positives is highly important in determining treatment course, especially in non-life-threatening disorders such as temporomandibular joint dysfunction (TMJD) [6]. Improving disc displacement criteria to better associate imaging findings to dysfunction should enhance the risk benefit ratio for TMJ dysfunction treatment.

On the other hand, the sensitivity of the alternative protocol is 30% less than that of the standard. Stated differently, of all subjects with arthralgia, 94% carried the diagnosis of ADD by standard measures, and 64% carried the diagnosis of ADD by alternative measures. This discrepancy stems from the alternative method's functional description of the disc position in determining derangement—a measure which is also more stringent—and in this cohort allows the subject to experience pain without a diagnosis of disc displacement. Realistically, the experience of TMJ pain is related to a myriad of causes, and internal derangement would not be expected to be the only one. Prior research on TMD has set “ideal” sensitivity and specificity levels at 70% [6, 21]. The standard protocol produced values of 94% sensitivity and 36% specificity, while the alternative protocol demonstrated 64% and 95%, respectively. Consequently, the latter provides a better and more appropriate compromise between sensitivity and specificity for identifying arthralgia in those with the ADD diagnosis and is consistent with the sensitivity (70%) and specificity (90%) goals established in the recent validation studies for the RDC for TMD [9].

The results highlight that, in employing the alternative criteria for diagnosing ADD, it is (i) unlikely to have a diagnosis of ADD without experiencing TMJ pain and (ii) further improbable to be without arthralgia but with ADD. While the standard method of diagnosis identifies the majority of subjects with arthralgia as having ADD, approximately 30% of its ADD patients are asymptomatic. Perhaps such a disc position is a variant of normal and should be considered adaptive rather than dysfunctional. Shaefer and Schiffman found that TMJ pressure-pain thresholds (PPTs) differed between asymptomatic TMJ arthralgia subjects with and without disc displacement with only those subjects without a history of disc displacement recording normal pressures [5]. A similar determination of PPTs in this study's subjects would allow insight into what degree of disc displacement correlates to normal versus mild dysfunction. Nevertheless, because the alternative protocol avoids false positives and is functionally based, it is more rigorous and clinically pertinent. It appears that the alternative criteria for determining disc displacement allow a more constructive assessment of TMJ anterior disc displacement.

While the results of this study are promising for better understanding the causes of TMJ arthralgia, there are limitations to our investigation. Because our study was retrospective, the imaging protocol for the MRIs could not be controlled. The images were collected from a variety of sources which may or may not have used the same techniques in creating the images. For example, there could be variation in patient factors such as head posture and closed mouth position (e.g., maximum intercuspation versus centric occlusion). The images may not have been taken using the same angulation through the condylar head (i.e., long axis), nor using the same number and size of slices or machine settings. As such, the central cut we used to interpret each MRI may not represent a uniform location in the TMJ. This could influence results, particularly considering that the disc position in the sagittal plane may be different depending on how medially or laterally in the joint the image is taken (see discussion in Rammelsberg's study). Furthermore, landmark identification may vary between and within examiners. A simple solution would be to perform a reliability study, wherein a sample of the MRIs that were interpreted for ADD is reinterpreted at a later date by the same examiner, and the diagnoses are compared to ensure concordance.

Another consideration in interpreting the images at one static point in time is the effect of treatment on the structures of the TMJ. Patients may have been on pre-, mid-, or posttreatment, of varying modalities. A number of studies have demonstrated alterations in the form and relative position of constituents of the TMJ due to orthopedic forces, for example, the use of functional appliances in orthodontics, which have been shown to influence the jaws by remodeling the condyle and glenoid fossa, repositioning the condyle, and autorotating the mandible [22]. Changes that could appreciably alter disc position include anterior or posterior movement of the condyle and flattening of the articular eminence [23–25]. For example, Wadhawan completed an MRI study demonstrating an initial posterior displacement of the disc during removable functional appliance therapy for class II malocclusion, but noted that the degree of displacement was not consistent with pathological derangement [22]. Furthermore, the disc position returned to the pretreatment location after functional appliance therapy was completed. Many of the studies relating to orthodontics and TMJ structures exhibit such reversal of the TMJ structure alterations over the long term [22–26]. Therefore, the position of the disc may vary according to the treatment undertaken, and the latter should be taken into consideration when analyzing the anatomy of the TMJ. This study also did not consider the presence or absence of osteoarthritic changes (OA findings) in the TMJ, nor synovitis, both factors which have been at least as well (if not better in the case of synovial tissue proliferation) associated to TMJ arthralgia as is disc displacement [9, 27, 28].

In order to address such limitations and advance the insight into TMJD etiology, a prospective investigation of anterior disc displacement would be of great benefit. Such a study would provide improved standardization of the MRI images and the classification of arthralgia and would allow for more variables to be included, such as the presence of joint inflammation, synovial tissue proliferation, and measures of specific inflammatory mediators [27–30]. Additional variables, in a temporal sequence, could aid in clarifying the effect disc displacement has on joint dysfunction and allow a better correlation of the degree of disk displacement to arthralgia symptoms [31]. A prospective study would offer a view into the evolution of TMJD over time and measure the influence and outcome of different therapies.

This investigation demonstrates a functionally and therefore clinically relevant method for diagnosing anterior disc displacement on MRI. Recently, Kalaykova et al. also promoted a more functional assessment to DD by using the anterior prominence of the head of the condyle rather than the 12 o'clock vertical to mark disk displacement [32]. Our protocol recommends utilization of the functional area of the articular eminence for imaging the TMJ by drawing a line perpendicular to the posterior slope of the articular eminence in the TMJ from which to determine anterior disk displacement in a sagital plane. This easy-to-use standard represents a step forward to the goal of understanding the etiology, progression, and treatment outcomes for temporomandibular joint dysfunction.

## Figures and Tables

**Table 1a:** (a)

Standard			
	Arthralgia	No arthralgia	Total no.
ADD	34	14	48
No ADD	2	8	10

Total no.	36	22	58

ADD: anterior disc displacement, arthralgia: a symptomatic TM joint, no arthralgia: an asymptomatic TM joint; total no. reflects the number of TM joint examined with that finding and diagnosis.

**Table 1b:** (b)

Alternative			
	Arthralgia	No arthralgia	Total no.
ADD	23	1	24
No ADD	13	21	34

Total no.	36	22	58

ADD: anterior disc displacement, arthralgia: a symptomatic TM joint, no arthralgia: an asymptomatic TM joint; total no. reflects the number of TM joint examined with that finding and diagnosis.

**Table 1c:** (c)

	Arthralgia	No arthralgia	Total no.
STD ADD	34	14	48
ALT ADD	23	1	24

Total no.	57	15	72

STD ADD: anterior disc displacement using the standard protocol, ALT ADD: anterior disc displacement using the proposed alternative protocol, arthralgia: a symptomatic TM joint, no arthralgia: an asymptomatic TM joint; total no. reflects the number of TM joint examined with that finding and diagnosis.

**Table 1d:** (d)

		STD		
		ADD	No ADD	Total no.
ALT	ADD	24	0	24
	No ADD	24	10	34

	Total no.	48	10	58

STD: standard protocol for determining disc displacement, ALT: proposed alternative protocol for determining disc displacement, ADD: anterior disc displacement, arthralgia: a symptomatic TM joint, no arthralgia: an asymptomatic TM joint; total no. reflects the number of TM joint examined with that finding and diagnosis.
